# Modeling Modulation of the Tick Regulome in Response to *Anaplasma phagocytophilum* for the Identification of New Control Targets

**DOI:** 10.3389/fphys.2019.00462

**Published:** 2019-04-18

**Authors:** Sara Artigas-Jerónimo, Agustín Estrada-Peña, Alejandro Cabezas-Cruz, Pilar Alberdi, Margarita Villar, José de la Fuente

**Affiliations:** ^1^SaBio, Instituto de Investigación en Recursos Cinegéticos IREC-CSIC-UCLM-JCCM, Ciudad Real, Spain; ^2^Facultad de Veterinaria, Universidad de Zaragoza, Zaragoza, Spain; ^3^UMR BIPAR, INRA, ANSES, Ecole Nationale Vétérinaire d’Alfort, Université Paris-Est, Maisons-Alfort, France; ^4^Department of Veterinary Pathobiology, Center for Veterinary Health Sciences, Oklahoma State University, Stillwater, OK, United States

**Keywords:** regulome, transcription, tick, *Ixodes scapularis*, *Anaplasma phagocytophilum*, ISE6 cells, vaccine

## Abstract

Ticks act as vectors of pathogens affecting human and animal health worldwide, and recent research has focused on the characterization of tick-pathogen interactions using omics technologies to identify new targets for developing novel control interventions. The regulome (transcription factors-target genes interactions) plays a critical role in cell response to pathogen infection. Therefore, the application of regulomics to tick-pathogen interactions would advance our understanding of these molecular interactions and contribute to the identification of novel control targets for the prevention and control of tick infestations and tick-borne diseases. However, limited information is available on the role of tick regulome in response to pathogen infection. In this study, we applied complementary *in silico* approaches to modeling how *Anaplasma phagocytophilum* infection modulates tick vector regulome. This proof-of-concept research provided support for the use of network analysis in the study of regulome response to infection, resulting in new information on tick-pathogen interactions and potential targets for developing interventions for the control of tick infestations and pathogen transmission. Deciphering the precise nature of circuits that shape the tick regulome in response to pathogen infection is an area of research that in the future will advance our knowledge of tick-pathogen interactions, and the identification of new antigens for the control of tick infestations and pathogen infection/transmission.

## Introduction

Ticks (Acari: Ixodida) are major vectors of pathogens affecting human and animal health worldwide, and consequently the focus of research for developing novel control interventions (de la Fuente, 2018). Among tick-transmitted pathogens, *Anaplasma phagocytophilum* (Alphaproteobacteria: Rickettsiales) is mainly transmitted by *Ixodes* spp. and the causative agent of human and animal anaplasmosis and tick-borne fever in small ruminants (Severo et al., 2015).

Recent developments in tick genomics have advanced research using latest omics technologies for the characterization of tick-host-pathogen interactions and the identification of candidate protective antigens (de la Fuente et al., 2016c,a, 2017; Gulia-Nuss et al., 2016; Shaw et al., 2017; de la Fuente, 2018). Vaccinomics, a holistic perspective based on the use of omics technologies and bioinformatics in a systems biology approach for the characterization of tick-host-pathogen molecular interactions is our platform for the identification of candidate vaccine antigens (de la Fuente and Merino, 2013; de la Fuente et al., 2016a, 2018; Contreras et al., 2017). In this context, tick cell lines constitute a valuable resource because it is a proven model for the study of tick-pathogen and particularly tick-*A. phagocytophilum* interactions, easy manipulation without animal experimentation, and the fact that *A. phagocytophilum* infects mainly one cell type in vertebrates (neutrophils) but multiple cell types in ticks better resembled by these cell lines (Munderloh et al., 1994; Severo et al., 2015; Villar et al., 2015; Bell-Sakyi et al., 2018).

The regulome (transcription factors-target genes interactions) and interactome (protein-protein physical and functional interactions) play a critical role in cell response to different stimuli including pathogen infection. Both regulome and interactome are implicated in transcriptional regulation, which is one of the most fundamental mechanisms for controlling the amount of protein produced by cells under different environmental and physiological conditions and developmental stages (Gronostajski et al., 2011; Vaquerizas et al., 2012; Shih et al., 2016; Rioualen et al., 2017). Therefore, the application of regulomics and interactomics to host/tick-pathogen interactions would advance our understanding of these molecular interactions and contribute to the identification of new control targets for the prevention and control of tick infestations and tick-borne diseases (de la Fuente et al., 2018; Artigas-Jerónimo et al., 2018a,b; Estrada-Peña et al., 2018).

Few studies have addressed the role of the regulome or regulon (part of the regulome including a set of genes that share a common regulatory element binding site) in the interaction between tick-borne pathogens and vertebrate hosts (i.e., Bugrysheva et al., 2015; Boyle et al., 2019). However, limited information is available on the role of tick regulome in response to pathogen infection (Artigas-Jerónimo et al., 2018b).

In this study, we applied complementary *in silico* approaches to modeling how *A. phagoctophilum* infection modulates tick vector regulome, and the possibilities for the identification of new control target antigens. This proof-of-concept research provided new information on tick-pathogen interactions and potential targets for developing interventions for the control of tick infestations and pathogen infection.

## Materials and Methods

### Datasets

The RNA sequencing (RNAseq) datasets of differential expression of *I. scapularis* transcription factors (TF) and target genes (TG) in response to *A. phagocytophilum* infection was obtained from previously published transcriptomics analyses in ISE6 cells, and fed adult female midguts and salivary glands (Ayllón et al., 2015; Villar et al., 2015). Gene ontology (GO) level-3 annotations for biological processes (BP) were conducted using Blast2GO software (version 3.0) ^1^ (Villar et al., 2014; Supplementary Dataset 1). The RNAseq data is available at https://doi.org/10.5061/dryad.50kt0 and http://www.ncbi.nlm.nih.gov/geo/query/acc.cgi?acc=GSE68881.

### Network Analysis of the Tick Regulome in Response to Infection

A network of interactions followed by a co-correspondence analysis (CoCA) was used for the integration of TF and TG interactions (regulome) of *I. scapularis* tick response to *A. phagocytophilum* infection. The methodology to build the network of interactions between proteins and functional metabolic processes has been previously described and validated (Estrada-Peña et al., 2018). This network consists of a set of nodes that are connected by edges where nodes are the interacting items, and links between nodes represent the strength with which they interact. In this development, a TF or TG is the source node and the cell metabolic process(es) in which it is involved are the target(s). The edge linking both nodes has a weight, which is the expression of either TF or TG. Networks were built separately for infected and uninfected *I. scapularis* ISE6 cells, salivary glands and midguts. Only TF and TG with GO functional annotations were included in the networks (Supplementary Dataset 1). Centrality is a fundamental property of a network because it refers to nodes that connect high score nodes (Opsahl et al., 2010; Estrada-Peña et al., 2018). In this context, “high score” applies to other nodes with high importance in the network. We calculated the importance of a node in the “traffic” between different nodes of the network using Betweenness Centrality (BNC), giving a higher score to a node that sits on many shortest paths of other node pairs (Barthelemy, 2004; Estrada-Peña et al., 2018). In our context, it is an indicator of the relative importance of a TF/TG in the links between two or more processes. Other calculated indexes included the PageRank (PR), a measure of the importance of the nodes linking with a given node, and the Weighted Degree (WD), which was calculated from the expression profile of each TF/TG linking to a cell process (Estrada-Peña et al., 2018).

The interactions between TF and TG were demonstrated using CoCA. Only TF/TG with values of BNC or PR higher than zero were included. We did the CoCA using the indexes of centrality obtained from the network explained above. The function “coca” of the package “cocorresp” was used for the R programming environment (Simpson, 2016). Data on BNC, PR and WD of each infected and uninfected datasets from ISE6 cells, salivary glands and midguts were entered into separate CoCAs. The analysis aimed to relate two different datasets from uninfected and infected samples to find patterns that are common to both and associating the TF and TG that are close in the reduced multivariate space and establishing correspondences. The plotting of the scores in the two first axes of the reduced space gives the interaction between TF and TG, i.e., the closer they are in the space, the higher is the expected interaction. The method produces a cloud of interacting TF and TG. To improve the resolution of the charts, we plotted only TF/TG that were at a maximum of two score units of distance. We assumed that other TG separated by more than 2 score units from the values of TF were not interacting with these TF.

### *In silico* Prediction of TF-TG Interactions

Putative DNA binding sites in TG for TF present only in infected tick ISE6 cells were predicted based on published information for TF-interacting sequences in other species, and the *I. scapularis* genomic scaffold whole genome shotgun sequence using cisTargetX^2^ and direct search for TF binding sequences in the predicted 5′ gene regulatory regions of the *I. scapularis* genome (Supplementary Dataset 2; Potier et al., 2012; Rougemont and Naef, 2012; Vaquerizas et al., 2012). The BP with higher representation in the upregulated than in downregulated regulome (peptidase inhibitor and stress response) in response to infection were selected for further characterization of TF-TG interactions.

### RNA Interference (RNAi) for Gene Knockdown in Tick ISE6 Cells

The *I. scapularis* ISE6 cells (provided by U.G. Munderloh, University of Minnesota, United States) was maintained in L-15B300 medium as described previously (Munderloh et al., 1994). Four different TF (HSF, Ap-2, Arx and Hox; Supplementary Dataset 2) were silenced using two siRNAs for each TF (HSF: 5′ GCA CUC AGG GCC AGG AUU A 3′ and 5′ CCU CGG AAG CAG ACA GGA A 3′; Ap-2: 5′ AGA AAG AGG ACA CGA AGA A 3′ and 5′ CCA AGA AAG AGG ACA CGA A 3′; Arx: 5′ CCA AGA AAG AGG ACA CGA A 3′ and 5′ GAC CGA AGC CAG AGU GCA A 3′; Hox: 5′ CCU CCA GCU UCA ACA CAU A 3′ and 5′ ACG CCA CGG CCG AGC UUA A 3′) provided by Dharmacon (GE Healthcare Dharmacon Inc., Lafayette, CO, United States). As control, two *Rs86* siRNAs (5′ CGG UAA AUG UCG AAG CAA A 3′ and 5′ GCG AAU AUG AAG UCG GUA A 3′) were used. The siRNA experiments were conducted by incubating ISE6 tick cells with 100 nM of each siRNA diluted in 100 μl of serum-free medium in 24-well plates using four wells per treatment. To facilitate siRNA transfection, DharmaFECT (GE Healthcare Dharmacon Inc.) was used following manufacturer’s recommendations. After 24 h, 0.5 ml/well of fresh medium was added. After 48 h of siRNA exposure, medium containing siRNA was removed and replaced with 1 ml fresh medium alone or containing cell free *A. phagocytophilum* NY18 obtained as previously reported (de la Fuente et al., 2005). Cells were incubated for a total of 72 h, and then collected for DNA and RNA extraction.

### Determination of Gene Knockdown and TG mRNA Levels by RT-qPCR

Total RNA was extracted from ISE6 cells using All Prep DNA/RNA/PROTEIN Mini Kit (Qiagen, Hilden, Germany) following manufacturer’s recommendations. Gene knockdown levels after TF RNAi were assessed for TF and TG by RT-qPCR on RNA samples using gene-specific oligonucleotide primers (Supplementary Table 1), the Kapa SYBR Fast One-Step RT-qPCR Kit (Kapa Biosystems, Roche Holding AG, Basel, Switzerland), and the QIAGEN Rotor-Gene Real-Time PCR Detection System (Qiagen). A dissociation curve was run at the end of the reaction to ensure that only one amplicon was formed and that the amplicons denatured consistently in the same temperature range for every sample. The mRNA levels were normalized against tick *rps4* using the genNorm method [Delta-Delta-Ct (ddCt) method] as described previously (Ayllón et al., 2013). Normalized Ct values were compared between test siRNAs-treated tick cells and controls treated with *Rs86* siRNA by Chi^2^-test (*p* = 0.05; *n* = 4 biological replicates).

### Determination of *A. phagocytophilum* DNA Levels by qPCR

Total DNA was extracted from infected cells using an All Prep DNA/RNA/Protein Mini Kit (Qiagen, Hilden, Germany). DNA samples were analyzed by qPCR using gene-specific primers for *A. phagocytophilum* *msp4* as previously described (Ayllón et al., 2013). Normalized against tick *rps4* Ct values were compared between test siRNAs-treated tick cells and controls treated with *Rs86* siRNA by Chi^2^-test (*p* = 0.001; *n* = 2–4 biological replicates).

## Results and Discussion

### Rationale and Experimental Design

The tick regulome in response to *A. phagocytophilum* infection was characterized in the *I. scapularis* tick vector to provide insights into tissue-specific regulome profiles, and the identification of potential targets for the control of tick infestations and pathogen infection/transmission. The experimental design included two independent methods for the *in silico* characterization of the tick regulome in response to *A. phagocytophilum* using transcriptomics data previously obtained from infected *I. scapularis* ISE6 cells, and fed female midguts and salivary glands (Supplementary Figure 1A). The first approach was based on a network analysis in which the nodes were either TF or TG together with their corresponding GO BP annotations, and the link between two nodes represented the expression of the gene (Supplementary Figure 1B). The indexes of centrality were calculated separately for each network of uninfected and infected samples, and only nodes of TF and TG with indexes of centrality higher than zero were used for co-correspondence CoCA analysis (Supplementary Figure 1B). The second approach was used in parallel with network analysis, and consisted in the *in silico* prediction of TF-TG interactions based on described TF recognition sequences by searching in the *I. scapularis* genomic scaffold whole genome shotgun sequence (Supplementary Figure 1C). This analysis was focused on TF present only in infected ISE6 cells, and TG in BP overrepresented in the upregulated than in the downregulated regulome in response to infection as a proof-of-concept to facilitate the identification of candidate target antigens for development of vaccines and other control measures. The results of the network analysis were then plotted with TF and TG together in the reduced space to demonstrate that the position of the TF correlates with the TG that are near to these TF after the CoCA (Supplementary Figure 1D). Finally, the results of both approaches were compared, and the TF-TG interactions predicted by both methods were functionally characterized by RNAi in *A. phagocytophilum*-infected and uninfected tick ISE6 cells (Supplementary Figure 1E).

### The *I. scapularis* Regulome Shows Tissue-Specific Signatures in Response to *A. phagocytophilum* Infection

**FIGURE 1:**
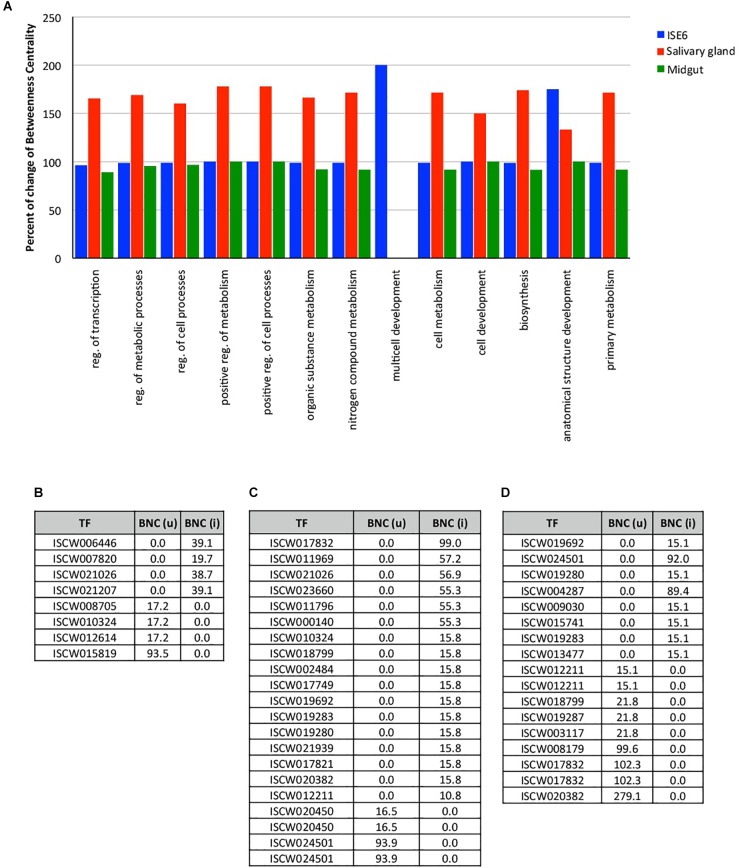
Changes in the expression of TF in tick ISE6 cells, salivary gland and midgut in response to *A. phagocytophilum* infection. **(A)** The percentage change of the index Betweenness Centrality (BNC) infection among uninfected and infected target organs in the 13 BP GO annotations. **(B)** Values of BNC of the 8 TF that showed the highest changes between uninfected BNC (u) and infected BNC (i) ISE6 cells. **(C)** Values of BNC of the 21 TF that showed the highest changes between uninfected BNC (u) and infected BNC (i) salivary glands. **(D)** Values of BNC of the 17 TF that showed the highest changes between uninfected BNC (u) and infected BNC (i) midgut. Abbreviations: reg., regulation; (u), uninfected; (i), infected.

For the construction of networks, a total of 144, 86, and 93 TF (Supplementary Dataset 3), and 5225, 3919, and 4341 TG (Supplementary Dataset 4) were used derived from tick ISE6 cells, salivary glands and midguts, respectively. Tick midgut did not show detectable differences in the network indexes of TF BP between uninfected and infected samples (close to 100% BNC; Figure 1A). However, the TF multicell development and anatomical structure development processes increased to near 200% in infected versus uninfected ISE6 cells (Figure 1A). The network centrality of all the TF processes showed a clear increase in infected salivary glands when compared to unifected controls (Figure 1A). The multicell development process was represented in TF from ISE6 cells only (Figure 1A).

Other than minor variations in the network indexes of the TF, each test showed different TF that were present or absent in either uninfected or infected samples. Four TF were detected only in uninfected ISE6 cells, and other 4 were recorded only in infected ISE6 cells (Figure 1B). The most prominent TF in ISE6 cells (ISCW01819) was completely inhibited in infected ISE6 cells (Figure 1B). The pattern was more complex in the salivary glands showing up to 17 TF recorded only in infected, and 4 in uninfected samples (Figure 1C). Nine TF were recorded only in uninfected and 8 only in infected midgut (Figure 1D). As in ISE6 cells, the three most highly represented TF in uninfected midgut were not recorded in infected samples (Figure 1D). The number of TG detected only in uninfected or infected samples varied from 197 (uninfected) to 206 (infected) ISE6 cells, 159 (uninfected) to 585 (infected) salivary glands, and 360 (uninfected) to 129 (infected) midgut.

**FIGURE 2:**
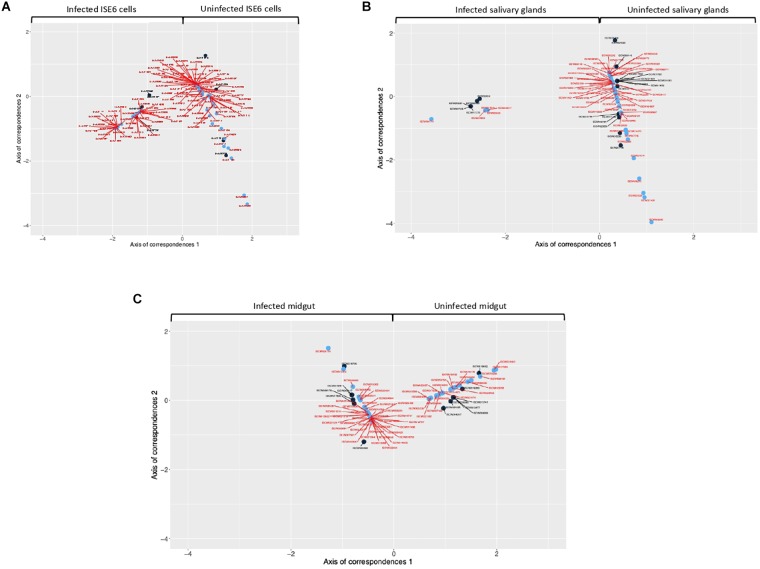
Co-correspondence analysis (CoCA) of TF and TG in uninfected and *A. phagocytophilum-*infected samples. CoCA was conducted in *I. scapularis* **(A)** ISE6 cells, **(B)** salivary glands and **(C)** midgut. The charts show the position of TF (black symbol and label) and TG (blue symbol and red label) after the CoCA of the indexes of centrality. The TF and associated TG with highest values of centrality in the network of infected cells appear together at negative values of the Axis 1 (*n* = 4, 4, and 9 in ISE6 cells, salivary glands and midgut, respectively). The TF and the associated TG with highest values of centrality in the network of uninfected cells appear together at positive values of the Axis 1 (*n* = 4, 17, and 8 in ISE6 cells, salivary glands and midgut, respectively). High-resolution images are shown in Supplementary Figures 2A–C.

Every detected TF that was unique for uninfected or infected samples was included in CoCA. Based on the values of network indexes of TG, 70 TG in uninfected and 88 TG in infected ISE6 cells, 5 TG in uninfected and 58 TG in infected salivary glands, and 43 TG in uninfected and 25 TG in infected midgut were included in the analysis. The results from multivariate analyses showed a clear correspondence between TF and TG recorded only in uninfected or infected samples while the origin of the first correspondence axis (value = 0) separated completely TF and TG occurring only in either uninfected or infected samples (Figure 2A–C and Supplementary Figures 2A–C). These analyses suggested that the TF closer to individual TG are likely to regulate the expression of these genes (Figure 2A–C and Supplementary Figures 2A–C).

**FIGURE 3:**
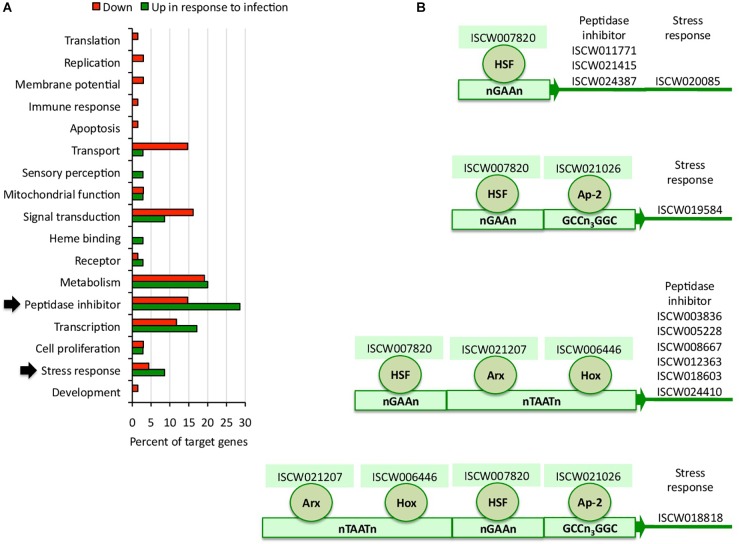
Biological processes affected by the tick ISE6 cells regulome in response to *A. phagocytophilum* infection. **(A)** Upregulated and downregulated target genes in the *in silico* predicted tick ISE6 cells regulome in response to *A. phagocytophilum* infection were grouped according to their BP. The BP with higher representation in the upregulated than in downregulated regulome in response to infection (arrows) were selected for characterization of TF-TG interactions. **(B)** Predicted regulatory DNA motifs according to regulatory factors identified by RNAseq in infected cells only and involved in the control of upregulated target genes annotated in the peptidase inhibitor and stress response BP with higher representation in the upregulated than in downregulated regulome.

These results evidenced tissue-specific differences between infected and uninfected cells, thus supporting previous findings at the mRNA, protein and metabolic levels in *I. scapularis* ISE6 cells, a model for hemocytes, midgut and salivary glands, which are involved in *A. phagocytophilum* life cycle in the tick vector (Ayllón et al., 2015; Villar et al., 2015; reviewed by de la Fuente et al., 2017). These results evidenced that the regulome regulates various BP involved in tick-*A. phagocytophilum* interactions (Figure 1A, 3A and Supplementary Dataset 2), a finding previously reported in other organisms (Shih et al., 2016; Casella et al., 2017).

### *A. phagocytophilum* Modulates the Tick Regulome to Upregulate Biological Processes That Facilitate Pathogen Infection

To complement the network analysis approach to tick regulome study, the putative DNA binding sites were characterized *in silico* for TF and TG in the upregulated regulome in response to infection in tick vector ISE6 cells (Figure 3A,B and Supplementary Dataset 2). In particular, the peptidase inhibitor and stress response BP with higher representation in the upregulated than in downregulated regulome (Figure 3A) were selected for further characterization of TF-TG interactions (Figure 3B).

**FIGURE 4:**
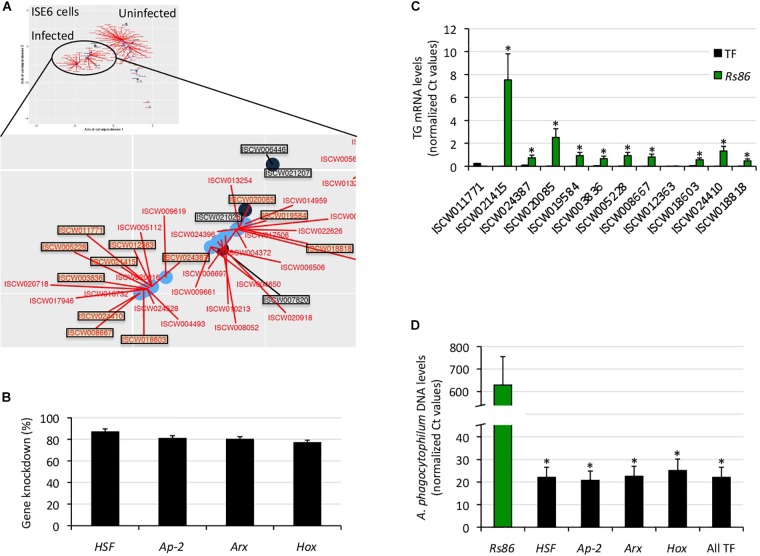
Functional characterization of selected TF-TG components of the tick ISE6 cells regulome. **(A)** The predictive results of network analysis and *in silico* prediction of TF-TG interactions were compared in infected ISE6 cells. The TF-TG interactions predicted by both methods (squared in black letter for TF and red letter for TG) were then functionally characterized by RNAi in tick ISE6 cells. **(B)** Percentage of TF gene knockdown with respect to Rs86 siRNA control in ISE6 cells. Normalized against tick *rps4* Ct values were compared between test siRNAs-treated tick cells and controls treated with *Rs86* siRNA by Chi^2^-test (*p* < 0.05; *n* = 4 biological replicates). **(C)** The TG mRNA levels were determined by qRT-PCR in ISE6 cells after TF gene knockdown or treatment with control *Rs86* siRNA. Normalized against tick *rps4* Ct values (average + S.E.) were compared between test siRNAs-treated tick cells and controls treated with *Rs86* siRNA by Chi^2^-test (^∗^*p* < 0.001; *n* = 4 biological replicates). **(D)** The *A. phagocytophilum* DNA levels were determined by qPCR in ISE6 cells after TF gene knockdown or treatment with control *Rs86* siRNA. Normalized against tick *rps4* Ct values (average + S.D.) were compared between test siRNAs-treated tick cells and controls treated with *Rs86* siRNA by Chi^2^-test (^∗^*p* < 0.001; *n* = 2–4 biological replicates).

The results showed a correlation between complementary *in silico* approaches (Figure 4A), therefore providing support for the network analysis of the regulome to predict at the transcriptomics level the most significant TF-TG interactions in response to stimuli such as pathogen infection.

To characterize the functional implications of selected TF-TG interactions predicted by both methodological approaches (Figure 3B, 4A), RNAi was used in ISE6 cells to knockdown the expression of TF and characterize the effect on TG mRNA and *A. phagocytophilum* DNA levels (Figure 4B–D). The results showed that after 77–87% (average ± S.D., 81 ± 4%) TF silencing (Figure 4B), the levels of all predicted TG except for ISCW011771 and ISCW012363 decreased when compared to *Rs86* siRNA-treated controls (Figure 4C). The two TG that were not downregulated after TF knockdown had the lowest mRNA levels (Figure 4C), which could affect the possibility of detecting differences between test and control cells. Alternatively, other TF or interacting proteins could be involved in the regulation of these genes. Nevertheless, except for ISCW011771 and ISCW012363 the results supported the prediction that these TF are implicated in the regulation of TG. Nevertheless, these TF-TG interactions should be corroborated in future experiments using different *in vitro* protein-DNA binding assays (Yang, 1998; Forde and McCutchen-Maloney, 2002; Deplancke and Gheldof, 2012; Ogawa and Biggin, 2012).

Regarding *A. phagocytophilum* infection, the results showed a 96–97% decrease in pathogen DNA levels after TF knockdown (Figure 4D). These results suggested that the TF and corresponding TG are upregulated by *A. phagocytophilum* to facilitate pathogen infection.

### Characterization of TF and Upregulated TG in Response to Infection as Putative Control Targets

The TF implicated in the regulation of selected TG included heat shock transcription factor (HSF), Ap-2, Aristaless-related homeobox gene (Arx) and Hox (Figure 3B). These TF has been described before to function in different transcriptionally regulated processes in other species. The mammalian Ap-2 TF has been shown to be involved in transcriptional activation and DNA binding/dimerization (Williams and Tjian, 1991). The HSF family has been implicated in the regulation of different physiological processes including cell response to stress and infection (Gomez-Pastor et al., 2018). Hox and Arx are members of a family of essential developmental regulators that bind to homeodomains target DNA sequences to regulate embryogenesis and neuronal processes in different organisms (Pellerin et al., 1994; Cho et al., 2012).

Few studies in other host-pathogen models have shown that some of these TF facilitate pathogen infection and therefore has been proposed as potential targets for control interventions. In primary peripheral blood monocytes, HSF1 is upregulated by human cytomegalovirus (HCMV) for pathogen survival, and has been suggested as a potential control target (Peppenelli et al., 2018). The tick-borne pathogen, *Ehrlichia chaffeensis*, upregulates the expression of certain Hox genes to facilitate infection through epigenetic mechanisms in human monocytic leukemia cells (THP-1) (Mitra et al., 2018). However, the role of these TF during pathogen infection in ticks has not been investigated before.

The BP modulated by the regulome of selected TF-TG interactions with a putative role in facilitating *A. phagocytophilum* infection included peptidase inhibitor and stress response (Figure 3A and Supplementary Dataset 2). The stress response upregulated TG included genes coding for peroxinectin and uncharacterized protein with heme binding and peroxidase activity (*n* = 2), and glutathione peroxidase with glutathione peroxidase activity. The peptidase inhibitor TG encoded for a carboxypeptidase inhibitor precursor with carboxipeptidase and metalloendopeptidase inhibitor activity, uncharacterized protein, Kunitz-type proteinase inhibitor 5 II, serpin-2 precursors and secreted salivary gland peptides with serine-type endopeptidase inhibitor activity (*n* = 6), and cystatin and salivary cystatin-L with cysteine-type endopeptidase inhibitor activity (*n* = 2).

These proteins are involved in key physiological processes during tick life cycle such as heme/iron metabolism and detoxification and tick-host interactions (Chmelar et al., 2016; de la Fuente et al., 2016a). Glutathione peroxidase belongs to the glutathione antioxidant defense system and protects eukaryotic cells from oxidative damage (Espinosa-Diez et al., 2015). Glutathione peroxidase levels and activity are affected in different ways by *Anaplasma marginale* infection in both vertebrate and tick cells (Reddy et al., 1988; More et al., 1989; Kalil et al., 2017; Esmaeilnejad et al., 2018). While the activity and expression of glutathione peroxidase and other components of the antioxidant system was lower in *A. marginale*-infected cattle and water buffaloes (Reddy et al., 1988; More et al., 1989; Esmaeilnejad et al., 2018), glutathione peroxidase coding gene was upregulated in embryonic *Rhipicephalus microplus* BME26 cells in response to *A. marginale* infection (Kalil et al., 2017). These results showed that *A. marginale* infection induces a differential response of the glutathione antioxidant defense system in the vertebrate and tick hosts. RNAi-mediated gene silencing of glutathione peroxidase and other antioxidant defense system genes increased *A. marginale* infection in BME26 cells, suggesting that the antioxidant response mediated by this molecule might play a role in the control of infection in ticks (Kalil et al., 2017). Another of the identified tick TG, peroxinectin, is a cell adhesion protein involved in melanization of pathogens in invertebrates (Sritunyalucksana et al., 2001; Cerenius and Söderhäll, 2004), was upregulated in crayfish resistant to white spot syndrome virus, and susceptible crayfish failed to upregulate this gene in response to viral infection (Yi et al., 2017). Strong cellular adhesion in response to the invading agent during crustacean encapsulation defense reaction was proposed as a protective mechanism mediated by peroxinectin in infected crayfish. The heme-binding lipoprotein (HELP), a transporter of heme in ticks, was previously found to be upregulated and downregulated in midguts and salivary glands, respectively, of *A. phagocytophilum*-infected ticks (Villar et al., 2016). HELP, together with Vitellogenin 1 and 2, was proposed to transport heme to other tick tissues such as salivary glands (Hajdusek et al., 2009). The uncharacterized protein with heme binding activity identified in this study may have a function similar to HELP, suggesting that *A. phagocytophilum* affects hemoglobin primary cleavage and heme transport in tick midguts and salivary glands, possibly to regulate the levels of heme in a tissue-specific manner with potential effects for pathogen and vector survival. The expression of Kunitz-type proteinase inhibitors have been found to be modified in several tick species in response to infection by tick-borne pathogens such as *Bartonella henselae* (Liu et al., 2014), flavivirus (McNally et al., 2012), *Babesia bigemina* (Antunes et al., 2012), and *A. marginale* (Zivkovic et al., 2010). Kunitz peptides are moonlighting proteins that perform multiple functions within the feeding lesion (Schwarz et al., 2014). Upregulation of Kunitz proteins in salivary glands of ticks infected with *B. henselae* (Liu et al., 2014) and flavivirus (McNally et al., 2012) may be associated with host immunity modulation at the feeding site. In contrast to upregulation, it is less clear why Kunitz peptides, including a Kunitz-type proteinase inhibitor 5, would be dowregulated in salivary glands following *A. marginale* infection (Zivkovic et al., 2010). Additional studies show that the expression of Kunitz peptides is complex and may be related to the tick and pathogen species (Rachinsky et al., 2007; Antunes et al., 2012).

These preliminary evidences based on selected TF-TG interactions support that the analysis of tick regulome in response to different stimuli such as pathogen infection could provide potential targets for the control of tick infestations and pathogen infection/transmission. Furthermore, some of these protein families have been proposed as protective antigens using a rational approach for the identification of tick vaccine protective antigens (de la Fuente et al., 2016a). As recently proposed (de la Fuente et al., 2018), the combination of regulomics with intelligent Big Data analytic techniques may contribute to the high throughput identification of candidate vaccine antigens.

## Conclusion

Our modeling of the modulation of the tick regulome in response to *A. phagocytophilum* infection provided new insights into the mechanisms that target specific functions in different tick tissues. These results supported the use of network analysis for the study of regulome response to infection. Although general mechanisms affected by *A. phagocytophilum* infection may be conserved even between tick and human cells (de la Fuente et al., 2016b), the effect of vector-pathogen co-evolution on pathogen isolates adaptation to grow in tick cells (Alberdi et al., 2015) may result in differences between isolates in the modulation of the tick cell regulome. Future research should be directed at validating the results of the network analysis for regulomics studies and the characterization of TF-TG interactions. Deciphering the precise nature of circuits that shape the tick regulome in response to pathogen infection is an area of research that in the future will advance our knowledge of tick-pathogen interactions, and the identification of new targets for the control of tick infestations and pathogen infection/transmission.

## Data Availability

All datasets generated for this study are included in the manuscript and/or the Supplementary Files.

## Author Contributions

MV, AE-P, AC-C, and JdlF conceived the study and designed the experiments. SA-J, PA, and MV performed the experiments. AE-P, MV, AC-C, and JdlF performed the data analysis. JdlF, SA-J, AE-P, and AC-C wrote the manuscript. All authors approved and contributed to the final version of the manuscript.

## Conflict of Interest Statement

The authors declare that the research was conducted in the absence of any commercial or financial relationships that could be construed as a potential conflict of interest.

## Footnotes

1 www.blast2go.com

2 https://omictools.com/cistargetx-tool
