# Supplementary material for: Modeling Modulation of the Tick Regulome in Response to Anaplasma phagocytophilum for the Identification of New Control Targets
Source: Front Physiol. 2019 Apr 18;10:462. doi: 10.3389/fphys.2019.00462 (PMC6482211; doi:10.3389/fphys.2019.00462)
Supplement: Supplementary file 5 [file Data_Sheet_1.PDF]

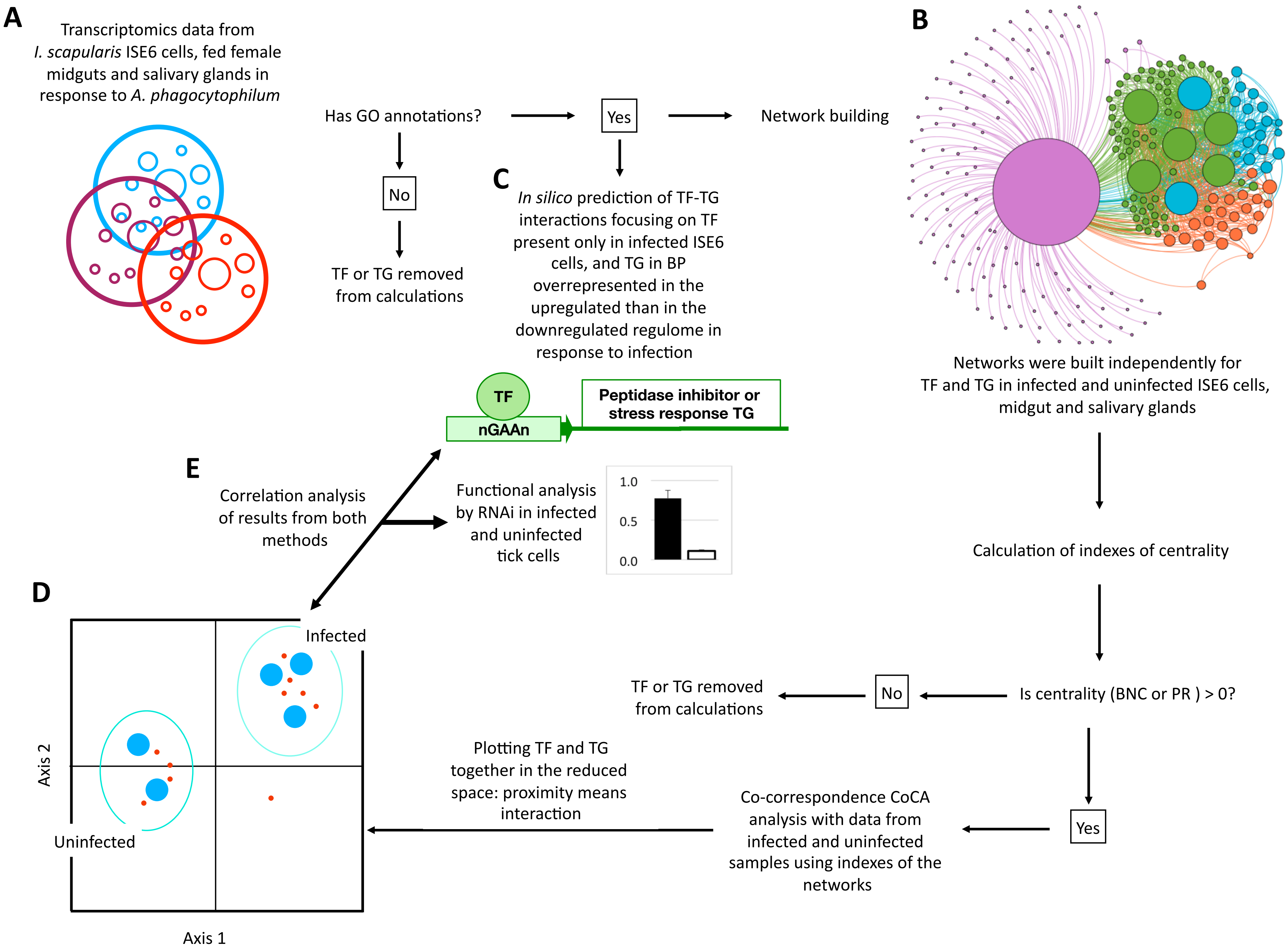

A
Transcriptomics data from *I. scapularis* ISE6 cells, fed female midguts and salivary glands in response to *A. phagocytophilum*
Has GO annotations?
Yes
Network building
No
TF or TG removed from calculations
C
In silico prediction of TF-TG interactions focusing on TF present only in infected ISE6 cells, and TG in BP overrepresented in the upregulated than in the downregulated regulome in response to infection
B
Networks were built independently for TF and TG in infected and uninfected ISE6 cells, midgut and salivary glands
TF
nGAAn
Peptidase inhibitor or stress response TG
E
Correlation analysis of results from both methods
Functional analysis by RNAi in infected and uninfected tick cells
1.0
0.5
0.0
Calculation of indexes of centrality
D
Infected
Uninfected
Axis 2
Axis 1
TF or TG removed from calculations
No
Is centrality (BNC or PR) > 0?
Plotting TF and TG together in the reduced space: proximity means interaction
Co-correspondence CoCA analysis with data from infected and uninfected samples using indexes of the networks
Yes

Supplementary Figure 1. Pipeline for the analysis of tick regulome in response to *A. phagocytophilum* infection. (A) Transcriptomics data were obtained by RNAseq of *I. scapularis* ISE6 cells, fed female midguts and salivary glands of uninfected and *A. phagocytophilum*-infected samples. (B) Only mRNAs with GO annotations were included in the building of a network in which the nodes are either TF or TG together with their corresponding GO BP annotations. The link between two nodes is the expression of the gene. After calculation of the indexes of centrality, separately for each network of uninfected or infected ISE6 cells, midgut and salivary glands, only nodes of TF and TG with indexes of centrality higher than zero were used for co-correspondence CoCA analysis. (C) In parallel, an *in silico* prediction of TF-TG interactions was conducted focusing on TF present only in infected ISE6 cells, and TG in BP overrepresented in the upregulated than in the downregulated regulome in response to infection. (D) The results of the network analysis were plotted with TF and TG together in the reduced space to demonstrate that the position of the TF correlates with the TG that are near to these TF after the CoCA. (E) Finally, the results of the network analysis were compared with those obtained by *in silico* prediction of TF-TG interactions, and those predicted by both methods were functionally characterized by RNAi in tick ISE6 cells.

Supplementary Figure 2A: ISE6 cells

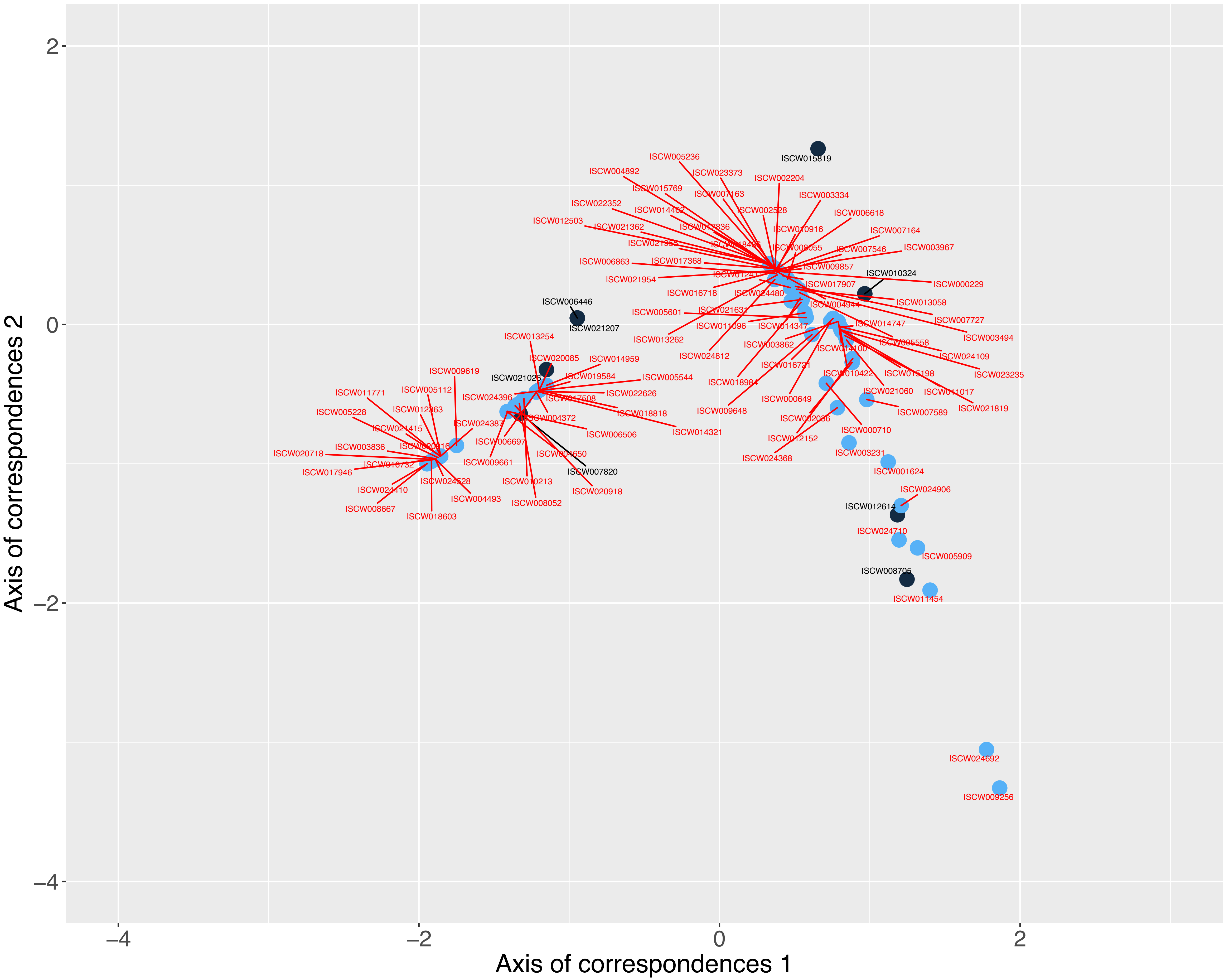

Supplementary Figure 2B: Salivary glands

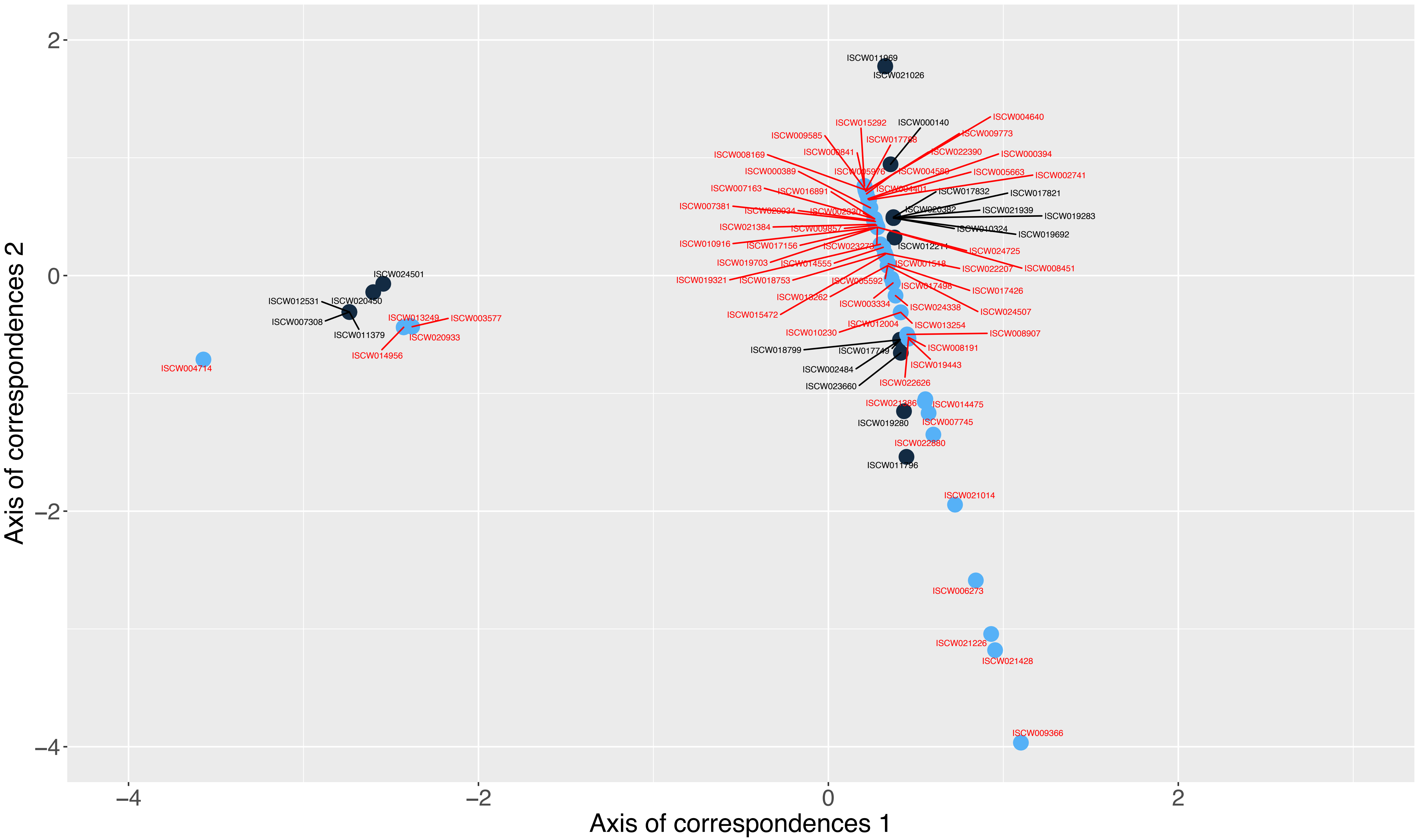

Supplementary Figure 2C: Midgut

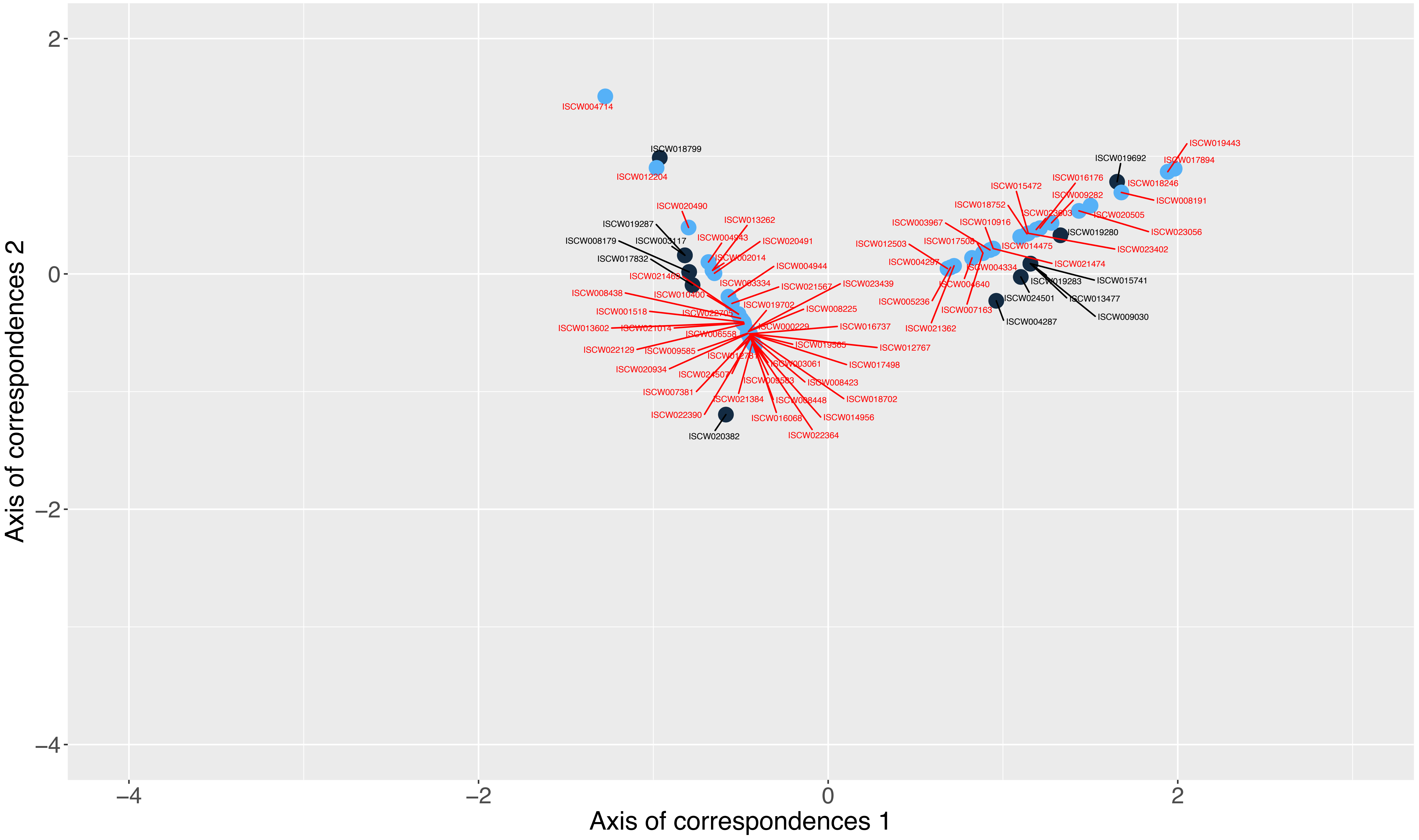

Supplementary Figure 2. Co-correspondence analysis (CoCA) of TF and TG in uninfected and *A. phagocytophilum*-infected samples. CoCA was conducted in *I. scapularis* (A) ISE6 cells, (B) salivary glands and (C) midgut. The charts show the position of TF (black symbol and label) and TG (blue symbol and red label) after the CoCA of the indexes of centrality. The TF and associated TG with highest values of centrality in the network of infected cells appear together at negative values of the Axis 1 (n = 4, 4 and 9 in ISE6 cells, salivary glands and midgut, respectively). The TF and the associated TG with highest values of centrality in the network of uninfected cells appear together at positive values of the Axis 1 (n = 4, 17 and 8 in ISE6 cells, salivary glands and midgut, respectively).

Supplementary Table 1. Oligonucleotide primer sequences for RT-qPCR analysis of TF and TG mRNA levels.

**TF**	**Forward primer (5' - 3')**	**Reverse primer (5' - 3')**
ISCW021207	TGGTTTCCGAGAGTCAGCAG	GACGTAACTCGGTCACGCTG
ISCW006446	GCGGTCAACGTGAATCCTCT	ACGAGGACATGGACTGGTCT
ISCW007820	CGAGTTACGACGCCCTCAAT	GGGATCCAGAAGTGCAACCA
ISCW021026	TGCTCAGTTCCACGTCCAAG	CGTGATGAGCGAGAAATGCG
**TG**	**Forward primer (5' - 3')**	**Reverse primer (5' - 3')**
ISCW011771	GACAAGATGGGCGTGTTCAG	GTTCATTCCAGCGACCACCT
ISCW021415	TCACGCGAGTCCTTATGGTG	TACAGCTAAGGCGTTCCACG
ISCW024387	TCTTCGGCTATTCCACGCTC	GAAGACAAGAGCCGCTGAGT
ISCW020085	CCTAACGCTGGCTGAAGTGA	TGTTCCTCCGAATGAGCTGG
ISCW019584	GAGTGCGCTCTACCACAAGT	CGTTCACGCTGATCTTGCTG
ISCW003836	AATTCTCGCAGGGGAACGAG	CCAGTTCAGGCGCTTCCTTA
ISCW005228	GCACTTCGGGCGTCTGTAAA	CTTGAAGGAGTTGTCGGCAC
ISCW008667	ACCTGACCCGAGTCATATCG	CTCGCAGTTTCGCAGTTCTC
ISCW012363	CGTACCTGCCGGAATTTGGA	GCCTTGAAACTCCTCGCACT
ISCW018603	TCACTTCGACACCGTAGCTG	GAAGGAGCTGACGGACTTGT
ISCW024410	AACGTGGCGATCTGGACATA	CCTCCGCAGTTTCTTTGGCA
ISCW018818	GACATTCGCGTGCATCATCG	CGTTGGCACAGAACAGCTTG

Full annotations for TF and TG with accession numbers shown here are included in Supplementary Dataset 2
